# Supplementary material for: Patient satisfaction of breast reconstructive surgery following mastectomy in Brunei
Source: PLoS One. 2023 Aug 23;18(8):e0289955. doi: 10.1371/journal.pone.0289955 (PMC10446170; doi:10.1371/journal.pone.0289955)
Supplement: Attachment — Submitted filename: Response to reviewers.docx [file pone.0289955.s001.docx]

PONE-D-22-31204

Patient Satisfaction of Breast Reconstructive Surgery following Mastectomy

PLOS ONE

| **Reviewer #1:** | **Author’s responses** |
| --- | --- |
| I have read with great interest the manuscript. Below my comments to the authors:  1. The “abstract” needs to be enriched with more information regarding the methodology and the results to make it clearer to the reader. 2. In the discussion section more studies with similar countries from other nations can be implemented (Persischetti et al DOI: 10.1007/s00266-022-02776-z). 3. In the material and methods and results section the data need to be mentioned in a more clear way. For example period of study is missing, patient characteristics, type of mastectomy and type of reconstruction etc. Please implement these information 4. Table 1 and 2 are incomplete and should be resubmitted. 5. References must be unified and edited as per journal recommendations. | 1. The methodology and results are detailed as per your comments. (Page 4 and 5) 2. This information is now added as per your comments (Page 19) 3. The requested information are now added (Page 7, 10, 11 and 12) 4. Table 1 and 2 reinserted after reformatting (Page 8 and 11) 5. References are now formatted according to the journal. |
| **Reviewer #2:** |  |
| The study is very interesting as it sheds light on patient-reported outcomes of breast reconstruction in a geographic area where this aspect of research has not been reported upon in a considerable manner so far. I commend the authors for their efforts. However, there are some areas of concern requiring some clarifications, revisions or corrections:  • Title: After carefully analyzing the contents of your manuscript, I would advise changing the title to the following: “Patient Satisfaction of Autologous Breast Reconstructive Surgery following Mastectomy in Brunei”. This addresses the population of patients you catered for more accurately and highlights the strengths of your content. • Abstract: Please note that your abstract is short (163 words) and does not address the number of patients recruited in the study, the period in which the study was conducted, the inclusion and exclusion criteria, etc. The maximum word limit according to the publication’s submission guidelines is of 300 words. Consider improving your abstract with the additional details and data that are relevant to the readership. • Introduction: Consider implementing how the mean age of patients undergoing mastectomy and breast reconstruction is of approximately of 50 years (PMID: 36376583), while survival of cancer patients is increasing, which supports the necessity for improved quality of life through breast reconstruction efforts (PMID: 35229192). • Materials and methods: Please implement the period of study (from 2012 until when?). Please describe inclusion and exclusion criteria more clearly. Did you exclude patients who underwent no reconstruction whatsoever or conserving breast surgery? Did you include all types of reconstructions? • Results: There are several points that should be raised and addressed: a) Please state more clearly that only 16 out of the 23 breast reconstruction patients were selected, and that only 14 out of 16 agreed to participate in the study. b) Additionally, I have some difficulty understanding which kind of reconstructive patients were recruited in the study. In your abstract you state that free TRAM, DIEP and implant-based reconstruction patients were included, but Table 2 includes only 14 patients out of which 4 received a TRAM (free?) and 9 received a DIEP flap. The total is 13, which means that something is missing or requires correction. This must be clarified. c) Were implant-based reconstructions not taken into consideration for this study? If not, it should be discarded from the abstract and should be implemented as an exclusion criteria. d) Consider implementing what kind of mastectomy the patients received. If a skin-sparing or radical mastectomy was selected, was the reconstruction assessed while having completed the reconstructive process? i.e. reconstruction of the nipple-areola complex (PMID: 33216178). • Discussion: I believe several aspects could be implemented to improve on its contents: a) Regarding abdominally-based breast reconstruction, you should consider implementing how DIEP flaps have been found to provide higher results in terms of satisfaction, particularly when associated with a nipple-sparing mastectomy, achieving the highest BREAST-Q scores in another study (PMID: 34559281). However, radiotherapy, chemotherapy, hormone therapy, age and BMI had no influence on patient satisfaction. b) In your study, 5 patients underwent adjuvant radiotherapy. Did it affect the volume of the reconstructed breast volumes in a significant manner? (PMID: 34272176) c) Consider discussing the efforts established in Brunei to improve early breast cancer diagnosis and to encourage breast reconstruction procedures in affected patients. d) Regarding the limitations, you should implement the reduced variability in terms of breast reconstruction procedures which featured only autologous options (DIEP, TRAM). • Consider implementing your references using a number instead of inserting the last name and year within the text. This makes tracking the references easier for the reader. Additionally, make sure you are using the Vancouver format for all your references (i.e. “Charmaz, K. (2014) Constructing grounded theory. sage.”, p. 17, Line 462). • Table 1 appears to be partially unreadable. Make sure you correct the formatting before resubmitting it. • Table 2 appears to be incomplete: while 4 patients underwent immediate reconstruction, you did not mention how many underwent delayed reconstruction. Additionally, were all immediate reconstructions free TRAMs? Were all the delayed reconstructions DIEP flaps? This could have caused a selection bias affecting patient-reported outcomes, and should also be mentioned in the study as a potential limitations. • As a minor observation, while the overall contents of your manuscript are clearly understandable, consider having your manuscript proof-read by a native English speaker to correct very small language mistakes and improve overall readability (i.e. “through providing adequate information” in Abstract, p.3 Line 85). | The title has been changed to “Patient Satisfaction of Breast Reconstructive Surgery following Mastectomy in Brunei”.  “Autologous” not used as we include breast implant surgery in our study too  This is now addressed and changes have been made accordingly. (Page 4 and 5 - Abstract)  This has now been addressed accordingly (Page 6)  This is now addressed. (Page 7 and 8)  a) This is now addressed. (Page 8)  b) This is now addressed. (See Table 2 Page 11)  c) This has now been addressed (Page 11 and 19).  d) This has been addressed (Page 17 and 18)  a) This has now been addressed (Page 18 and 19)  b) This is now added in discussion. (Page 17)  c) This is now added in discussion. (Page 16 and 17)  d) This is now added. (Page 20)  This is now addressed.  Table 1 and 2 reinserted after reformatting (Page 8 and 11)  This is now added in discussion. (Page 18 and 19)  The paper was checked by native English speaker before resubmission. |
