# Supplementary material for: Patient satisfaction of breast reconstructive surgery following mastectomy in Brunei
Source: PLoS One. 2023 Aug 23;18(8):e0289955. doi: 10.1371/journal.pone.0289955 (PMC10446170; doi:10.1371/journal.pone.0289955)
Supplement: Attachment — Submitted filename: 2.Response to reviewers_R2.docx [file pone.0289955.s002.docx]

PONE-D-22-31204R1

Patient Satisfaction of Breast Reconstructive Surgery following Mastectomy

PLOS ONE

| **Reviewer # 2** | **Author’s responses** |
| --- | --- |
| This is a revision to a previously assessed manuscript. The changes which have been made to the title, abstract, manuscript and tables are satisfactory. Well done. Some minor corrections are however still warranted: • The abstract has been significantly improved upon, but now exceed the maximum word limit of 300 (it now counts 333 words): please trim it down a little. • I advise against using “Plastic Reconstructive Surgery” as an acronym. It has been repeated 6 times throughout the manuscript. It is best to spell it out in full or to use “our department” instead. • You still cited Cano et al. (2013) (Materials and methods, p. 8) in the previous reference style. Please use Vancouver style and number the reference. • Please add the name of the company, the city and country of its headquarters next to the name of software and other copyrighted material. For example: Q-Score© (The Q Scores Company, Manhasset, New York, USA); RStudio© (RStudio Inc, Boston, MA, USA). • Type of mastectomy has been successfully implemented in the discussion, however it needs to be mentioned in the Materials and Methods section as well. • Since all patients received a non-nipple sparing mastectomy, you mentioned that some opted to receive nipple reconstruction while others did not. Has this data been recorded? Additionally, which techniques did you use? Consider referencing this paper by Paolini et al. for added background and a comprehensive description of techniques for nipple-areola complex reconstructions. (PMID: 33216178). • Regarding abdominally-based breast reconstruction, please implement findings from this study from Santanelli di Pompeo et al. according to which patients were more satisfied with breast reconstruction when they received a DIEP flap compared to implants, particularly when associated with a nipple-sparing mastectomy, as demonstrated by higher BREAST-Q scores (PMID: 34559281). This is worthwhile mentioning in your discussion because no patient could receive a nipple-sparing mastectomy in your study for oncological reasons. | - Abstract word count has been reduced to 300 words. - “PRS” changed to “our” department (page 5,6,7,14,16,20) - Cano et al. (2013) added - Added on page 7 - Non nipple-sparing mastectomy included in Materials & Methods on page 6. - Number of patient opted for nipple recon added. - C-V flap technique used - Paper on C-V flap added as reference. Jalini et al (p19) - Paper (Di Pompeo et al) included and referenced (p19) |
| **Reviewer #3** |  |
| Thanks for the opportunity to review this study. This is study on an interesting subject for Brunei context. I am glad that the satisfaction and quality of life of women that undergo a breast reconstructive surgery following mastectomy was assessed. Important points are made. However, there are some points I would like to raise mainly around writing of the manuscript. See below my suggestions.  Abstract: 1. The abstract is very long and scattered. The authors should try to summarise the results of their study more concisely, considering the 300 words.  Introduction: 2. The part concerning the quality of life of these patients should be explored in more detail, highlighting which aspects are most affected and what is the experience of women undergoing BRS both before and after surgery. The authors could include further literature investigating this issue.  Method: 3. It is not clear who conducted the individual interviews or led the groups? Was it a psychologist, a mental health professional, or a doctor? The authors should clarify this. 4. What kind of open-ended questions were asked to obtain the qualitative data? The authors should state the questions. 5. The topics touched upon in the interview may have had an important resonance on the emotionality of the patients. Were they guaranteed appropriate psychological support in case they needed it? 6. The part on the analysis of the thematic data and the protocol used should be further investigated. It would be interesting to understand how the authors arrived at the themes and sub-themes presented in the study.  Results: 7. The authors should specify how many patients did an individual interview and how many chose to be in a group. This could be an important variable that could have an influence on the answers.  Discussion, limits and conclusion: 8. The discussions appear to be very long, dispersive, and not very clear in order to generate linear thinking with the results found and provide more insights. The authors should improve and implement these aspects. 9. It would be interesting to explore further the discussion regarding the results that emerged between patients who underwent imminent reconstruction and those who underwent it at a later step. 10. Authors could discuss and elaborate on the clinical implications of their results. 11. This study is characterised by the assessment of the satisfaction of patients undergoing BRS and their quality of life only to the post-operative phase. For this reason, the authors cannot report any improvement in the quality of life and in the psychological health of the patients. There is no longitudinal part that can confirm this. The authors should correct their manuscript (abstract, discussion, conclusions) specifying that high quality of life scores was found nowadays and not improvements. 12. The authors should be careful to claim that there has been an improvement in psychological health. This only seems to emerge to some extent from the qualitative analyses. Psychological health was not investigated quantitatively and through specific outcomes such as depression, anxiety, or psychological symptoms in general. The authors should review this aspect and consider whether to include it as a limit. 13. The authors should include as a limitation the fact that the study participants underwent reconstruction surgery at different times, and some are also awaiting further procedures to improve the aesthetic outcome. This might have influenced the results regarding the assessment of quality of life and satisfaction. | - Abstract word count has been reduced to 300 words. - Added into Introduction - Reaby LL to added on page 5 - SN is the primary researcher and a doctor in PRS department - Table 2 Interview guide table inserted (page 8) - Psychological support added. - Two researchers spent a large amount of time iteratively reading and understanding the transcripts before performing coding and formulation of themes and subthemes. Several rounds of member checking was conducted to ensure discrepancies are minimized. Constant comparative method was used after each interview transcript to enhance analysis. Final themes and subthemes were formed with consensus and no major disagreement among researchers. (p8) - All opted for individual interviews (page 12) - Discussion has been improved - Added in Discussion - Abstract, Discussion and Conclusion amended - Included as limitation - This limitation has been included   Thank you for your kind review and valuable comments. |
