# Supplementary material for: Patient satisfaction of breast reconstructive surgery following mastectomy in Brunei
Source: PLoS One. 2023 Aug 23;18(8):e0289955. doi: 10.1371/journal.pone.0289955 (PMC10446170; doi:10.1371/journal.pone.0289955)
Supplement: Attachment — Submitted filename: 2.Response to reviewers_R3.docx [file pone.0289955.s003.docx]

PONE-D-22-31204R2

Patient Satisfaction of Breast Reconstructive Surgery following Mastectomy

PLOS ONE

| **Reviewer # 2** | **Author’s responses** |
| --- | --- |
| This is a revision to a previously assessed manuscript. While I am satisfied with the changes to what has been implemented beforehand, I believe that some changes are still needed regarding the formal aspects of your qualitative study’s methodology: • I find that the defined objectives and research questions of your qualitative study are not expressed clearly enough, and they should be stated more explicitly at the end of your manuscript’s introduction.  • Please make sure you implement the COREQ checklist, or other relevant checklists listed by the Equator Network, such as the SRQR, to ensure complete reporting (http://journals.plos.org/plosone/s/submission-guidelines#loc-qualitative-research). You may specify in your manuscript’s methods which checklist was used.  • While you described participants’ selection and recruitment, the description of the sampling strategy, including rationale for the recruitment method, participant inclusion/exclusion criteria and the number of participants recruited is still lacking and should be improved upon.  • Data collection procedures are not detailed enough. For instance, was the assignment of a PIN done as a randomization strategy to guarantee respondents’ privacy? How did you assess quality of data? Did you take into account consistency of collection and completeness?  • The implementation of Table 2 improves your manuscript greatly. However, your data analysis procedures must be described in further detail to enable replication.  • Finally, please identify and discussion any potential source of bias, and add any missing limitation related to your methodology at the end of your discussion. | Thank you for your kind review and valuable comments.  Aim and research questions provided in page 6 at the end of last paragraph  COREQ checklist was implemented (Added in Page 6)  Amended on Page 6. There were only 23 patients in total that had undergone BRS since services started in 2012.  Page 7. PIN given to ensure confidentiality & privacy.  Page 9. Added on quality of data, consistency and completeness of data collection.  Page 10. Data analysis procedures described in details  Amended on Page 20. |
| **Reviewer #3** |  |
| Thank you to the authors for addressing most of the concerns expressed above. I have further points to bring to their attention below:  1.“These reasons are common amongst women wearing external prostheses following mastectomy in regaining a semblance of femininity” Is this statement made by the authors in reference to their patients or is it generalisable to all women undergoing reconstruction with implants? If it is in reference to all, the authors should include the citation.  2.As suggested above, it would be interesting to explore in the introduction the psychological aspects of these women's quality of life, both pre and post reconstructive surgery. Several studies in the literature could be used, such as that of Fanakidou et al. (doi: 10.1007/s11136-017-1735-x) or Howard-McNatt (doi: 10.2147/BCTT.S29142). The authors should further investigate these aspects.  3.In the introduction all the variables that the authors will later investigate with their study should be presented. For this reason, a part should be devoted to the sexual well-being of women as this aspect is then discussed in the results. The authors should add this part and revise the whole introduction making sure to present the variables of interest adequately and thoroughly.  4.The discussion should be deepened with respect to the results that emerged on psychological well-being, sexual well-being, and general quality of life. This should be done by comparing with other studies and highlighting the clinical implications. | Thank you for your kind review and valuable comments.  Edited to “our patients” on Page 5.  Added referenced Howard-McNatt (Ref 11).  Fanakidou has now been added in Page 4.  There were no specific variables we intended to investigate individually. All outcomes were derived from the Breast-Q questionnaires and the interview guide from Klassen et al. Amendments added on Page 5.  Amendments added on Page 19. |
